# Performance Analysis of Segmentation and Classification of CT-Scanned Ovarian Tumours Using U-Net and Deep Convolutional Neural Networks

**DOI:** 10.3390/diagnostics13132282

**Published:** 2023-07-05

**Authors:** Ashwini Kodipalli, Steven L. Fernandes, Vaishnavi Gururaj, Shriya Varada Rameshbabu, Santosh Dasar

**Affiliations:** 1 Department of Artificial Intelligence & Data Science, Global Academy of Technology, Bangalore 560098, India; 2 Department of Computer Science, Design, Journalism, Creighton University, Omaha, NE 68178, USA; 3 Department of Computer Science, George Mason University, Fairfax, VA 22030, USA; vgururaj@gmu.edu; 4 Department of Computer Science & Engineering, Global Academy of Technology, Bangalore 560098, India; shriyavr52@gmail.com; 5 Department of Radiologist, SDM College of Medical Sciences and Hospital, Dharwad 580009, India; drsantoshdasar@gmail.com

**Keywords:** ovarian tumours, UNet, convolutional neural networks, VGG 16, DenseNet, ResNet, Dice score, Jaccard score

## Abstract

Difficulty in detecting tumours in early stages is the major cause of mortalities in patients, despite the advancements in treatment and research regarding ovarian cancer. Deep learning algorithms were applied to serve the purpose as a diagnostic tool and applied to CT scan images of the ovarian region. The images went through a series of pre-processing techniques and, further, the tumour was segmented using the UNet model. The instances were then classified into two categories—benign and malignant tumours. Classification was performed using deep learning models like CNN, ResNet, DenseNet, Inception-ResNet, VGG16 and Xception, along with machine learning models such as Random Forest, Gradient Boosting, AdaBoosting and XGBoosting. DenseNet 121 emerges as the best model on this dataset after applying optimization on the machine learning models by obtaining an accuracy of 95.7%. The current work demonstrates the comparison of multiple CNN architectures with common machine learning algorithms, with and without optimization techniques applied.

## 1. Introduction

Ovarian cancer stands out as a commonly diagnosed type of cancer worldwide. Considering the fact that it usually goes unrecognised until it reaches terminal stages, ovarian cancer is a leading reason for high mortality rates among women as a gynaecological illness. Ranking fifth in deaths due to cancer among women, the risk of being diagnosed with ovarian cancer peaks between the ages of 55 and 64, on average [1]. Silent symptoms and undetermined causes act as major factors for late diagnosis and ineffective screening methods.

The American Cancer Society claims that around 19,710 women will be diagnosed with ovarian cancer and that around 13,270 deaths will occur from ovarian cancer in 2023 in the United States [2]. In the past few years, significant developments in the field of biomedical imaging have contributed to the domain of cancer detection. With interdisciplinary approaches being popularized to solve objectives, Medical Imaging can be combined with Machine Learning and Deep Learning disciplines to effectively detect and categorize tumours. Ultrasound and CT scan images contain large amounts of information, making them ideal to use in the case of the implementation of Deep Learning algorithms.

The symptoms are not only vague in nature, such as bloating, abdominal pain, fatigue, etc., but they are also noticeable only in the later stages. The lack of symptoms showing up early often leads to delayed medical examination of the subject and therefore late detection of the tumour in general. Also, unlike in a few other cancers, there is no ascertained screening process for ovarian cancer. The most common approach is to have the subject undergo a scanning process, usually a CT scan, where the radiologist would go through hundreds of images and determine if there is a tumour present or not. Not only is this time-consuming, but also prone to error due to possible false positives. All of the above reasons can be considered as the main challenges to detect ovarian tumours. The advent of technology like Deep Learning mechanisms can aid in the detection of a tumour by increasing its efficiency in terms of time and accuracy.

This paper aims to provide a comparative study of the detection and classification of ovarian tumours using Machine Learning and Deep Learning algorithms, using CT images of ovaries. Multiple ML models and CNN variants were used for this purpose and the comparison was carried out inter-categorically as well as intra-categorically. The Literature Survey section covers the latest developments and ongoing research, not only in the detection of ovarian cancer but also in how state-of-the-art Deep Learning algorithms are used in other medical scenarios.

The organization of the paper is as follows: Section 2 reports the literature survey of the latest research performed in the area of biomedical imaging and of several learning algorithms used. Section 3 demonstrates the methodology, along with the steps and the models used in the current work. Section 4 presents the experimental results and the discussion. Section 5 provides the concluding thoughts.

## 2. Literature Review

### 2.1. Medical Imaging Classification Using CNN

Jung et al. [3] used ultrasound images of the lower body region of females to remove unwanted information in the frame and classify the ovaries into five classes—normal, cystadenoma, mature cystic teratoma, endometrioma, and malignant tumour. They used a texture-based analysis for tumour detection and trained a convolutional autoencoder or CNN-CAE. The images before and after the autoencoder are both fed into CNNs such as Inception, ResNet and different variants of DenseNets. Weighted class activation mapping or Grad-CAM was used to visualise the result. It can be noted that the model classified better when unnecessary data were removed using CNN-CAE. DenseNet121 and DenseNet161 were the better performers amongst all the algorithms used when parameters like accuracy, sensitivity, specificity, and the area under the curve (AUC) were considered to be metrics of performance.

Wang et al. [4] used pelvic CT scan images to detect and segment out ovarian cancer tumours simultaneously, i.e., creating a multi-task deep learning model. They proposed a model called YOLO-OCv2, which was an enhancement of their previously proposed algorithm. Mosaic enhancement was also used here, in order to improve the background information of the object. However, the multitask model YOLO-OCv2 outperformed other algorithms like Faster-RCNN, SSD and RetinaNet, which were trained on the COCO dataset. In this work, Mahmood et al. [5] created a Nuclei segmentation model that could be used to segment out the nuclei in multiple locations of the body. The authors used Conditional Generative Adversarial Networks, or cGAN, as they can control the GAN training output depending on a class. The model was trained on synthetically generated data along with real data in order to make sure that sufficient input was present. The model was trained with data from nine organs and was tested on four organs, where it outperformed its peers, such as FCN, U-Net and Mask R-CNN. Guan et al. [6] used mammographic images to detect breast cancer using CNN models. The authors focused on Affine transformations and synthetic data generation using GANs.

### 2.2. Medical Imaging Classification Using Ensemble Deep Learning

According to Karimi et al. [7], a Vision Transformers (ViT) algorithm was proposed which divided images into Image Patches. The proposed algorithm using transformers did not use any convolution operations to segment the brain cortical plate and the hippocampus in MRI images of the brain. The results were compared with FCN architectures like 3D UNet++, Attention UNet, SE-FCN and DSRNet. The proposed network performed segmentation accurately when compared to the other, and with a significantly smaller number of labelled training images. Xu et al. [8] worked on histopathological whole-slide images (WSIs) to detect ovarian cancer using CNNs trained on images of multiple resolutions. The authors proposed a new modified version of ResNet50 called the Heatmap ResNet50 algorithm for CNN-based patch selection, and ResNet18 along with MR-ViT was used for ViT-based slide classification. Li et al. [9] introduced a variation of UNet known as CR-UNet to simultaneously segment out ovaries and follicles from transvaginal ultrasound (TVUS) images. The proposed model was then compared with models like DeepLabV3+, PSPNet-1, PSPNet-2 and U-Net to find that the proposed model outperformed them all. In the proposed work by Goodfellow et al. [10], an adversarial net framework was suggested that loosely resembles a minimax two-player game. Nagarajan et al. [11] and Zhao et al. [12], in their research work, provided three approaches that were used to classify ovarian cancer types using CT images. The first approach used a deep convolutional neural network (DCNN) based on AlexNet, which did not provide satisfactory results. The second approach had an overfitting problem. To overcome this, GAN was used in the third approach to augment the image samples along with the DCNN, which provided the best results out of the three approaches in metrics such as precision, recall, f-measure and accuracy. The research work of Saha et al. [13] included a novel 2D segmentation network called MU-net, which was a combination of MobileNetV2 and U-Net used to segment out follicles in ovarian ultrasound images. An USOVA3D Training Set 1 dataset was used. The proposed model was evaluated against several other models from previous works in the literature, and was shown to be more accurate, with an accuracy of 98.4%. Jin, J et al. [14], in their work, used four UNet models: U-net, U-net++, U-net with Resnet and CE-Net to perform automatic segmentation. In Thangamma et al. [15], the k-means algorithm and fuzzy c-means algorithm were used on ultrasound images of ovaries. It was concluded that the fuzzy c-means algorithm provided a better result than the k-means algorithm The work by Hema et al. [16] involved FaRe-ConvNN, which applied annotations on the image dataset, where the images had three categories: epithelial, germ and stroma cells. In order to avoid overfitting and other issues due to the small dataset size, image augmentation using image enhancement and transformation techniques like resizing, masking, segmentation, normalization, vertical or horizontal flips and rotation was undertaken. FaRe-ConvNN was used to compensate for manual annotation. After the region-based training in FaRe-ConvNN, a combination of SVC and Gaussian NB classifiers was used to classify the images, which resulted in impressive precision and recall values [17]. In the works carried out by Ashwini et al. [18,19,20], various Deep Learning models were used to segment the CT scanned images and classify them using variants of CNN. In the work [18,19], Otsu’s method was used to segment the tumour and a dice score of 0.82 and Jaccard score of 0.8356 were obtained. Further, to perform segmentation, cGAN was used [20] and, in this study, the segmentation and classification of tumours were carried out in a single pipeline, which obtained the dice score of 0.91 and the Jaccard score of 0.89. Similarly, in the works carried out by Fernandes et al. [21,22], according to the work [21], the authors proposed the segmentation of brain MRI images using entropy-based techniques. As per [22], the detection and classification of brain tumours by parallel processing was carried out using big data tools such as Kafka and PySpark.

### 2.3. Deep Learning in Medical Imaging Segmentation

Koonce et al. [23] shed light on EfficientNet, which comprised the inverted residual blocks of MobileNet v2 combined with the MnasNet architecture to form a robust model for performing Image Recognition. Rehman et al. [24] at BU-Net used a Residual Extended Skip (RES) block and a Wide Context (WC) block in a U-Net architecture to implement the proposed model, BU-Net, to segment Brain tumour cells in MRI scanned images. In the current work by Rehman et al. [25], the authors proposed a model named BrainSeg-Net to achieve the segmentation of tumour. The proposed model included a Feature Enhancer (FE) block at every encoder stage to protect critical information that could be tampered with during the convolution and transformation processes. Jalali et al. [26] proposed ResBCDU-Net for lung segmentation in CT images, which was used in applications such as in detecting lung cancer. To form the ResBCDU-Net, a pre-trained ResNet-34 network was used in place of an encoder in a typical U-Net model. The proposed method performed better than models like U-Net, RU-Net, ResNet34-UNet and BCDU-Net when measured using several evaluation metrics. Maureen et al. [27] and Neelima et al. [28] carried out an extensive review of bone image segmentation by considering the methods used in medical additive manufacturing. According to this review, global thresholding is the most commonly used method for segmentation and has obtained an accuracy of under 0.6 mm. Further, the authors have proposed using other advanced thresholding methods that may improve the accuracy to 0.38 mm. In the work carried out by Minnema et al. [29], the CNN-based STL method was applied for bone segmentation in CT scan images, which was able to accurately segment the skull and obtain a mean dice value of 0.92 ± 0.4. As per [30], a residual spatial pyramid pooling (RASPP) module was proposed to minimize the loss of location information in different modules. On similar lines, the work proposed by [31] optimized the CNN UNet model by applying it on a CT dataset generated from the MRI images. The results showed that the model performed well on the CT images when compared with the MRI images.

## 3. Methodology

### 3.1. Dataset Description

The current research work was carried out in collaboration with SDM Dharwad Hospital, Dharwad, Karnataka, India. The dataset used in the research work was obtained from SDM Dharwad College and Hospital. The entire work has been approved by the ethical approval committee of the hospital. The radiologist annotated the lesions, which acted as the ground truth for the study. The size of the dataset is as follows: there were 2560 benign and 2370 malignant images. For the complete study of the tumours, all three orientations of the images were considered—axial, coronal and sagittal views. The images were 2D.

### 3.2. Segmentation Using U-Net Model

**Figure 1:**
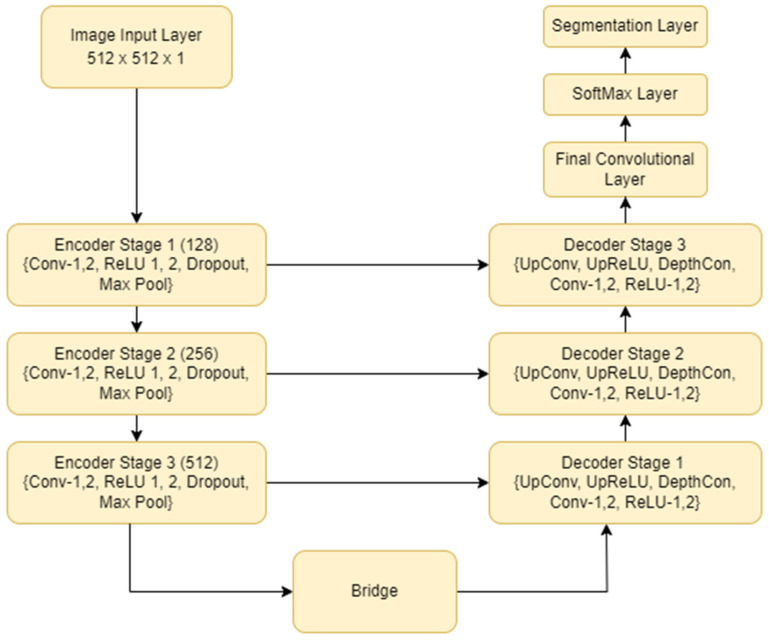
U-Net model architecture for segmentation of benign and malignant tumours.

U-Net is one among the oldest image segmentation models, first introduced in the paper U-Net: Convolutional Networks for Biomedical Image Segmentation. It comprises an encoder–decoder architecture for down-sampling and up-sampling, respectively. The U-Net [32] architecture is displayed in Figure 1.

The connections between encoder and decoder were the skip connection, which concatenated the encoder feature map with the decoder. This helped in the training process due to the backward flow of gradients. Image segmentation could be assumed as a combination of classification and localization tasks. The skip connections and decoder networks constituted the important aspects of the U-Net. The encoder network, also known as the contracting network, learned a feature map of the input layer. The task of the encoder was very similar to a classification task, in identifying the objects present in the image. In between the encoder and decoder networks lies the bottleneck layer, which comprised two convolutional layers followed by the ReLU activation layer. This layer produced the final feature map representation. The decoder network, also called the expansive network, took the feature map, as outputted by the bottleneck layer, as an input and outputted a segmentation mask. This was accomplished with the aid of skip connections. The task of the decoder is mainly to localize the object in the image. Skip connections, indicated with the grey arrows in the architecture, used contextual features learned from the encoder block and generated the segmentation map.

### 3.3. Detailed Methodology

**Figure 2:**
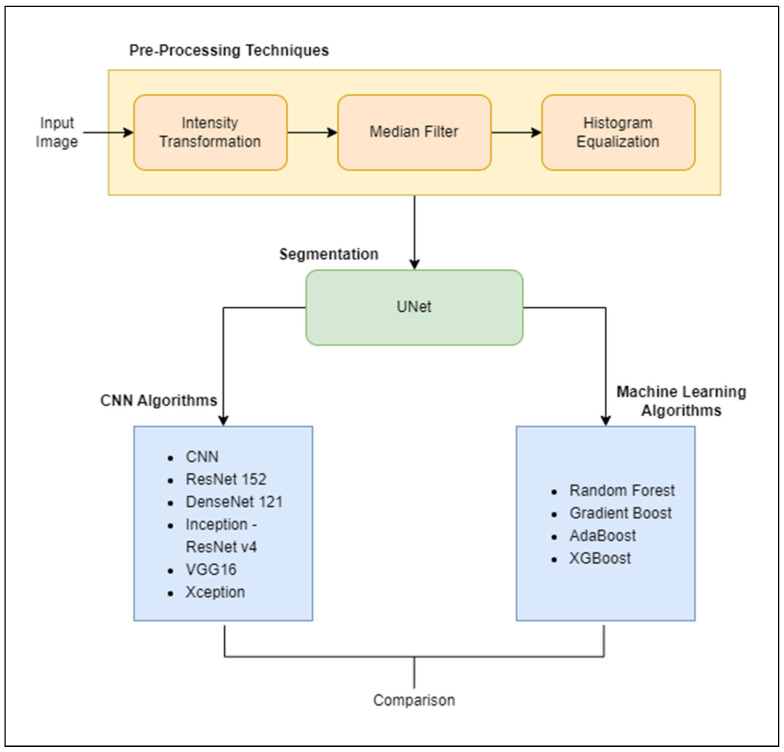
Flow of the implementation.

Figure 2 describes the implementation flowchart. The CT scan images of the ovarian region collected were used as input images for the algorithms. These images underwent pre-processing techniques before they were fed into these models as input. Pre-processing includes a series of digital image processing techniques to support the segmentation of the input image. Intensity transformation was performed to obtain higher-quality images, by enhancing the pixel intensity of the image. Median filter was applied to reduce speckle-like patterns formed by noise in the CT scanned image and for better edge detection. Histogram equalization helped in highlighting fine details in the image to segment out the Region of Interest (ROI), which in our case was the ovarian tumour, with ease.

After undergoing the pre-processing steps, the images were fed as input to a UNet model for segmenting out the tumour in the CT scanned image. UNet [32] is a Convolutional Neural Network (CNN) architecture applied to medical images to perform segmentation tasks. It is mainly used to segment images with complex shapes and sizes, such as tumours. With its increasing popularity in biomedical imaging, it is also being used in other fields such as satellite imaging, etc. The segmented images were then used as input in several deep learning CNN variants and machine learning models for classification. The input instances were classified into two categories, namely, benign and malignant tumours, to detect whether the subject is at risk of Ovarian cancer or not. The CNN models implemented included the simple CNN model, ResNet 152, DenseNet 121 and Inception—ResNet v4. VGG16 and Xception. These are the state-of-the-art models. The reason for choosing smaller layers of these models was due to small dataset available for this research work. Machine learning models like Random Forest, Gradient Boost algorithm, AdaBoost and XGBoost were also used. The outcomes of all these algorithms were then compared.

## 4. Results and Discussions

The results section is divided into three parts. The first section describes the segmentation results using UNet, the second section describes the classification results using variants of CNN and the third section shows the comparison of the deep learning results with machine learning results.

### 4.1. Experimental Settings

Using bicubic interpolation, all the images were resized to 512 × 512 pixels. The batch size was set to 64 and every model was trained up to 1000 epochs. A final fully connected layer with ReLU activation function had 256 hidden neurons, followed by a dropout layer with a probability of 0.5 to prevent overfitting. Adam optimizers were used with the parameter values (beta 1 and beta 2) set to 0.6 and 0.8 and the learning rate as 0.0001. The last dense layers of all architecture were modified to output two classes corresponding to benign and malignant. All pre-trained CNN models were fine-tuned separately. The training and testing of the proposed architecture was implemented using Python using the keras package and run on Nvidia RTX 3060 GPU with 32 GB RAM.

### 4.2. Segmentation Results Using the UNet Model

The performance metrics used to evaluate the segmentation process were *Dice* and *Jaccard* scores. The loss function used in the UNet was Softmax.

*Dice* score: This metric was used to determine the similarity between two images. Here, two images refer to the ground truth and the segmented image. The equation for the *Dice* score is given below:

$$Dice=\frac{2*\left|S\;\cap G\right|}{\left|S\right|+\left|G\right|}$$

where *S* indicates the segmented region that needs to be evaluated and *G* indicates the ground truth of the image. | | indicates the cardinality of the set. The *Dice* score always has a value between 0 and 1. The greater the value, the better the segmentation.

*Jaccard* score: This metric is used to calculate the overlap area between the segmented and the ground truth. The equation for the *Jaccard* score is given below:

$$Jaccard=\frac{dice}{2-dice}$$

The evaluated values of the *Jaccard* score range lie between 0 and 1; the greater the value, the better the segmentation results obtained.

**Figure 3:**
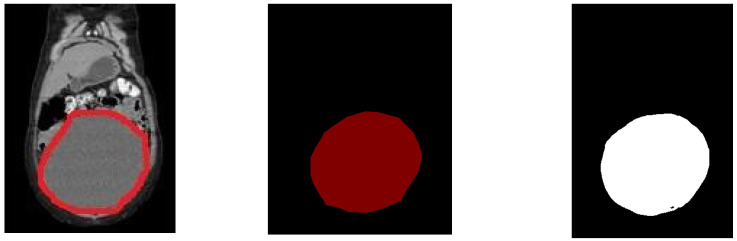

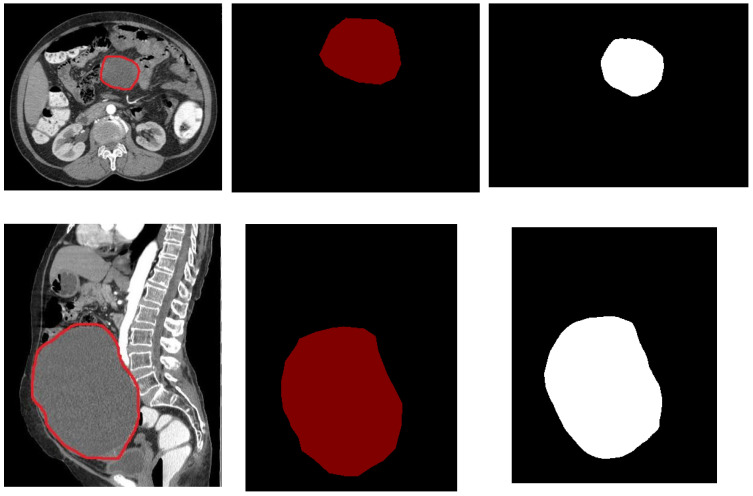
Benign dataset. Towards the (**left**) is the input image, the (**middle**) is the label and the (**right**) is the segmented image.

Figure 3 presents the images of the input, ground truth (label) and the segmented images for benign images. It is observed from Table 1 that UNet performed well for the benign images, as the shape of the tumour was well-grown and the borders were clear. Table 1 has the 20 sample results from the testing set.

From Figure 3, it can be observed that the ground truth and the segmented images were very close and the same is reflected in the Dice and the Jaccard score tabulated in Table 1. The column “class0” indicated the background and the column “class1” indicated the tumour. The average Dice score was 0.998 ± 0.12 for class0 and 0.981 ± 0.19 for class1. The average Jaccard score was 0.995 ± 0.22 for class0 and 0.964 ± 0.20 for class1.

**Figure 4:**
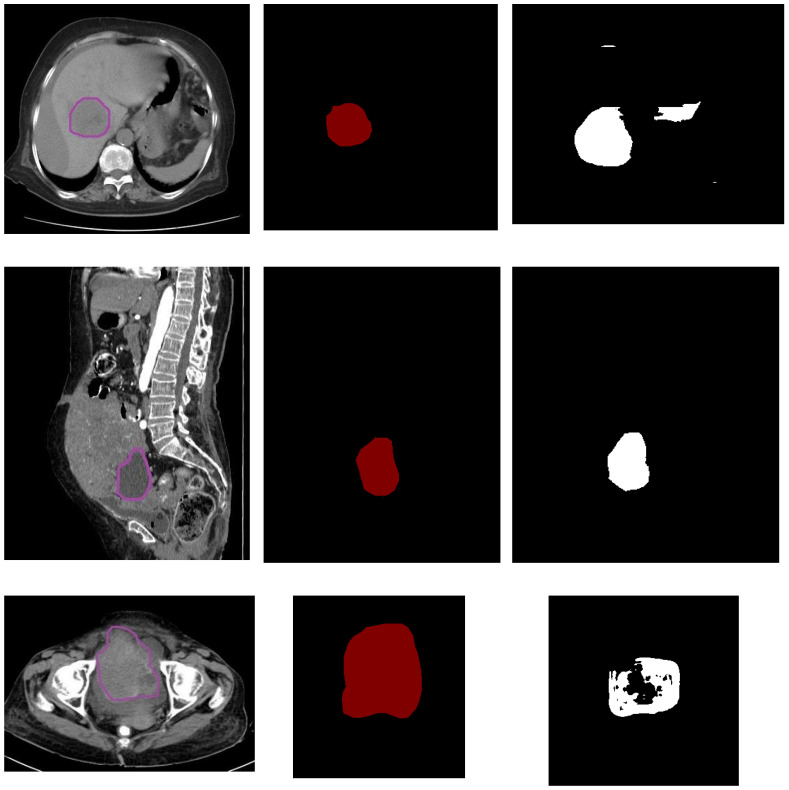
Malignant dataset. Towards the (**left**) is the input image, the (**middle**) is the label and the (**right**) is the segmented image.

Figure 4 presents the images of the input, ground truth (label) and the segmented version of malignant images. It can be observed from Table 2 that UNet did not perform well in detecting malignant instances, as the shape of the tumour was very uneven and small and the borders were very uncertain due to the characteristics of the malignant tumours. Table 2 has 20 sample results from the testing set.

From Figure 4, it can be observed, from the malignant image in the first row, that the tumour size was very small and that the pixel intensity is much less. Due to size and intensity, the UNet model performance was low, with the dice and Jaccard score as follows: class0_dice was 0.98 and class1_dice was 0.87, and class0_jaccard was 0.99 and class1_jaccard was 0.84.

On similar lines, in the malignant image present in the middle row, we observed that the tumour had grown fully and the intensity of the pixel was high. Due to this, UNet performed well, with the Dice and Jaccard scores as follows: class0_dice was 0.996 and class1_dice was 0.950, and class0_jaccard was 0.993 and class1_jaccard was 0.926. The malignant image in the last row shared a similar characteristic to the first row image. The tumour was very uncertain with respect to size, shape and border. Hence, lower values were obtained in the segmentation results.

Table 2 shows 20 sample image performances from the test dataset. Due to the nature of the malignant tumours’ size, shape, border and intensity of the pixels, the performance of UNet on malignant tumours was overall less when compared to the performance on benign tumours.

**Figure 5:**
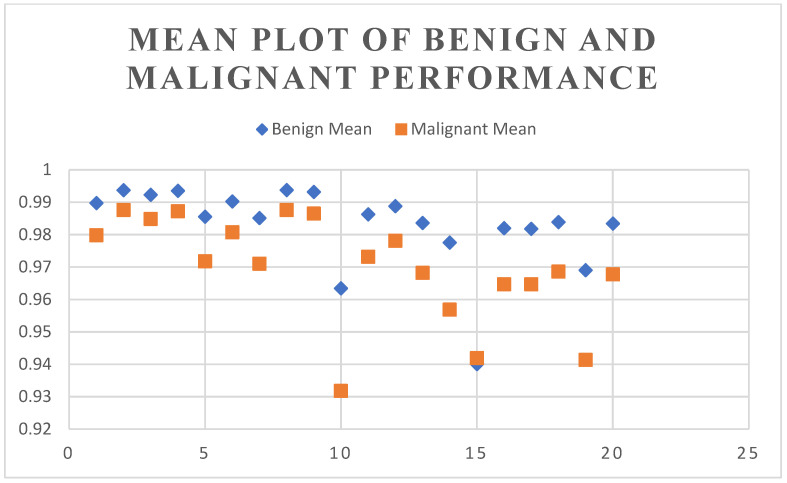
Mean plot of benign and malignant performance.

**Figure 6:**
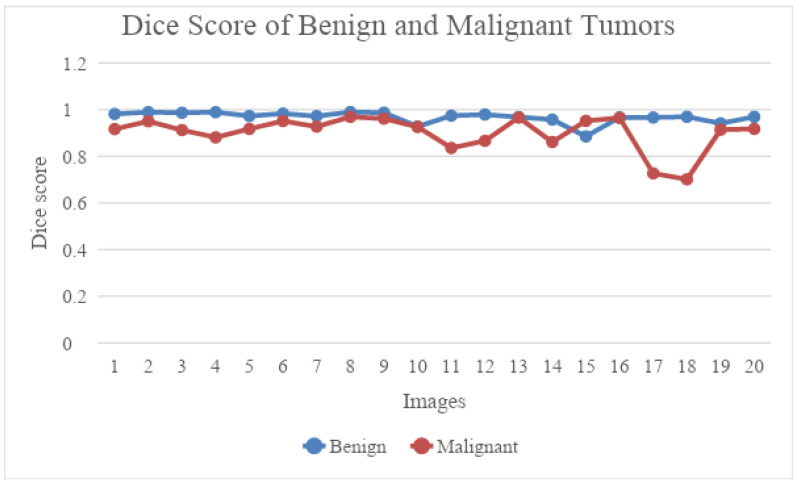
Dice score comparison between benign and malignant tumours.

**Figure 7:**
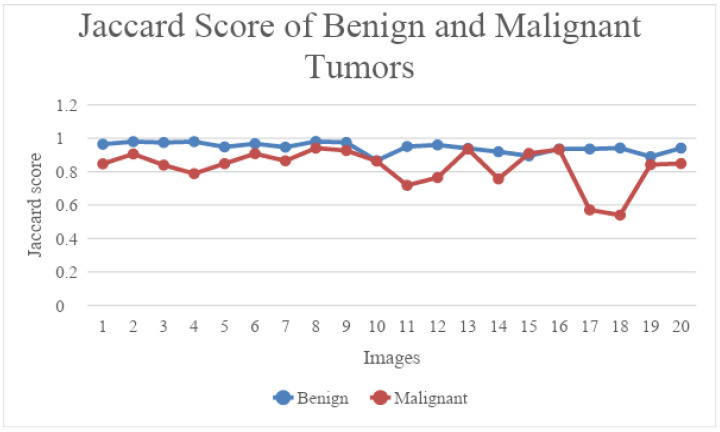
Jaccard score comparison between benign and malignant tumours.

The mean of Table 1 and Table 2 is shown in Figure 5. From the graph below, depicted in Figure 6, we can see that UNet performed well on benign images when compared to malignant images. The dice score range for benign images was between 0.992 and 0.998, whereas the dice score for the malignant category ranged between 0.70 and 0.91. Figure 7 shows the Jaccard score comparison for benign and malignant images.

### 4.3. Classification Results Using Variants of the CNN Model

Since the current research work is in the medical domain, it is extremely important to measure *TP*, *TN*, *FP* and *FN* and take appropriate measures for the necessary treatment. The different performance metrics used to analyse the classification results in our experiment were *accuracy*, *precision*, *recall* and *F1 score*. The metrics were expressed mathematically, as follows:

$$Accuracy=\frac{TP+TN}{TP+TN+FP+FN}\times 100$$

$$Precision=\frac{TP}{TP+FP}\times 100$$

$$Recall=\frac{TP}{TP+FN}\times 100$$

$$F1\;score=\frac{2\times Precision\times Recall}{Precision+Recall}$$

**Figure 8:**
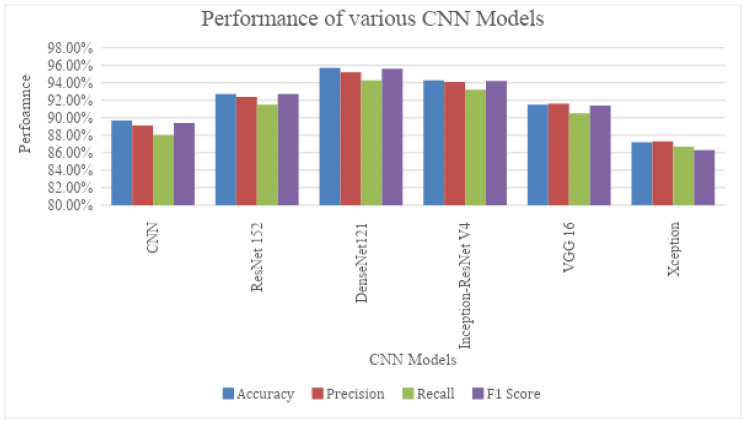
Diagrammatic representation of the performance of the CNN models.

From Table 3 and Figure 8, it was observed that DenseNet121 outperformed the other CNN models used. Owing to the connectivity patterns in the DenseNet architecture, the information would not be lost or vanished by the time it reached the last layer.

**Figure 9:**
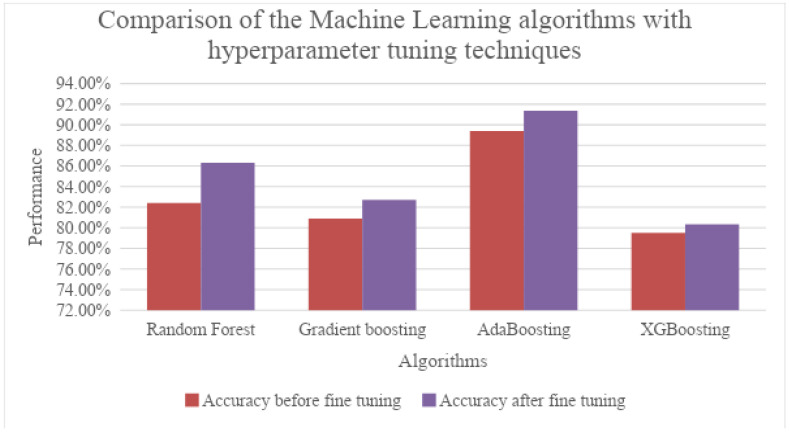
Diagrammatic representation of the comparison of the results with tuning techniques.

The results were then compared with ensemble machine learning models using the same data, for classification. From Table 4, it can be noted that AdaBoosting outperformed the other ensemble machine learning models. From Table 5 and Figure 9, it can be observed that the performance of the learning algorithms was improved with the tuning techniques.

The different optimization techniques used for fine tuning the parameters were HyperOpt, Optuna and Multi-Fidelity Optimization. After fine tuning the parameters, the machine learning algorithms’ performances had improved.

HyperOpt works as follows: When compared with other hyperparameter techniques like randomised search and grid search, HyperOpt reduces the number of trials to find the best parameters by defining the search algorithm which selects the best input values from each new iteration. The key feature of the Optuna hyperparameter is its automated search for optimized hypermeters even in the large search space by pruning the unpromising trails for faster results. This technique also has a unique characteristic of parallel search, which makes searching faster. The multi-fidelity technique is well suited when the model is very large or when the size of the dataset is very large.

After fine tuning the parameters, the improved results using ML algorithms are as follows.

## 5. Conclusions and Discussion

Ovarian cancer is one of the most dangerous diseases found in women. An alarming number of deaths are caused every year due to the diagnosis of ovarian cancer only in the third and fourth stages. Advancements in the field of deep learning have helped to solve several issues in the medical field, as well. Although a completely dependable solution, we can find a significant amount of work carried out in helping doctors and radiologists to detect tumours.

The findings of this work can be generalised and extended to other clinical settings, but only with datasets having similar characteristics, for example, to detect the presence of tumours in other organs of the body, like breast mammographic, whose images detect breast cancer, or MRI or CT scans to segment follicles or parts of the brain using MRI images, nuclei cell segmentation, etc., but only after undergoing the process of fine tuning of parameters. Regarding the concept of transfer learning, deep learning models can increase the performance. By means of transfer learning, the efficiency of the models implemented in other cases can be improved, as it would leverage the knowledge gained by being trained on a larger and more diverse dataset. Using ensemble architectures could also improve the accuracy and robustness of the model. However, it is important to note that there are a few limitations to extending the work. The type of data, the objective to be achieved and the environment of the experiment could be very different from the current work. The resolution of the images, noise levels and parameters considered in consolidating the dataset also affects the performance. The type of model used in the experiment would also be one of the factors.

These computer screening methods take CT scan images of the ovarian region as input and perform pre-processing, segmentation and classification on them, using various algorithms. In the current work, multiple algorithms in machine learning, as well as in deep learning fields, have been implemented and compared. Segmentation was carried out using the UNet model. A total of 20 images were sampled from both the categories—benign and malignant—on which the UNet algorithm was applied. The performance was measured using the Jaccard score and Dice score. It was found that the model performed better when segmenting benign tumours. The UNet model did not perform well for the extremely small tumours, as seen in the malignant results. Therefore, more complexed model such as Transformers can be tried for the segmentation of smaller and uncertain tumours.

Deep learning models include several variants of CNNs such as CNN, ResNet 152, DenseNet 121, Inception-ResNet V4, VGG16 and Xception. The evaluation metrics used are accuracy, precision, recall and F1-score. It was observed that DenseNet 121 surpasses the other CNNs in all the metrics, while scoring an accuracy of 95.70%. The machine learning algorithms involved were Random Forest, Gradient Boosting, AdaBoosting and XGBoosting. The same metrics used above were used to evaluate these models, as well. Values obtained clearly showed that AdaBoosting outperformed the other Machine Learning algorithms considered, scoring an accuracy of 89.40% and leading the other metrics as well.

To boost the performance of the machine learning models, hyperparameter tuning was performed using HyperOpt, Optuna and Multi-Fidelity Optimization techniques. The resulting values were significantly better than those sans optimization. However, AdaBoosting remained the best performing technique among the ML models considered. We noted that deep learning architectures performed more efficiently than machine learning models with optimization techniques when classifying benign and malignant tumours in the CT scanned images, which proves them to be an aiding tool.

This experiment can be used as a Computer Aided Detection (CAD) system which helps doctors and radiologists by narrowing the region of the tumour present, segmenting it precisely and classifying the tumour into benign and malignant categories. The person in charge could use this as an assisting tool, as it would reduce effort and time while increasing accuracy.

## Figures and Tables

**Table 1:** Dice score and Jaccard score for benign tumour.

Image	Benign			
	Class0_Dice	Class1_Dice	Class0_Jaccard	Class1_Jaccard
CT_1	0.99756	0.98193	0.99514	0.96451
CT_2	0.99775	0.98975	0.99552	0.97972
CT_3	0.99758	0.98710	0.99518	0.97454
CT_4	0.99738	0.98974	0.99479	0.97969
CT_5	0.99777	0.97334	0.99556	0.94807
CT_6	0.99701	0.98348	0.99404	0.96751
CT_7	0.99761	0.97269	0.99523	0.94684
CT_8	0.99729	0.99024	0.99459	0.98068
CT_9	0.99895	0.98740	0.99790	0.97512
CT_10	0.99882	0.92817	0.99765	0.86597
CT_11	0.99786	0.97470	0.99577	0.95066
CT_12	0.99814	0.97956	0.99637	0.95994
CT_13	0.99862	0.96864	0.99724	0.93920
CT_14	0.99718	0.95798	0.99439	0.91935
CT_15	0.99460	0.88555	0.98926	0.89461
CT_16	0.99696	0.96709	0.99395	0.93623
CT_17	0.99686	0.96678	0.99375	0.93569
CT_18	0.99777	0.96998	0.99553	0.94171
CT_19	0.99643	0.94171	0.99290	0.88984
CT_20	0.99724	0.96965	0.99450	0.94101

**Table 2:** Dice score and Jaccard score for malignant tumour.

Image	Malignant			
	Class0_Dice	Class1_Dice	Class0_Jaccard	Class1_Jaccard
CT_1	0.99485	0.917209	0.989753	0.847079
CT_2	0.996533	0.950731	0.993091	0.906089
CT_3	0.994843	0.912769	0.989738	0.839535
CT_4	0.996412	0.881644	0.992849	0.788339
CT_5	0.998503	0.9179	0.99701	0.848258
CT_6	0.99661	0.951691	0.993242	0.907835
CT_7	0.997797	0.927598	0.995603	0.864972
CT_8	0.997502	0.969651	0.995017	0.941091
CT_9	0.996653	0.961937	0.993328	0.926665
CT_10	0.995213	0.926677	0.990471	0.863373
CT_11	0.983532	0.836238	0.967598	0.718565
CT_12	0.995118	0.866793	0.990284	0.764902
CT_13	0.999053	0.966729	0.998107	0.9356
CT_14	0.995317	0.861702	0.990677	0.757008
CT_15	0.997093	0.952554	0.994203	0.909406
CT_16	0.997117	0.965229	0.99425	0.932794
CT_17	0.995457	0.727209	0.990954	0.57135
CT_18	0.986163	0.701614	0.972704	0.540374
CT_19	0.998494	0.914519	0.996992	0.842501
CT_20	0.998775	0.918228	0.997552	0.848818

**Table 3:** Performance of various CNN in classifying ovarian tumours.

Sl. No	CNN Architectures	Accuracy	Precision	Recall	F1 Score
1.	CNN	89.7%	89.1%	88.0%	89.4%
2.	ResNet 152	92.7%	92.4%	91.5%	92.7%
3.	DenseNet121	95.7%	95.2%	94.3%	95.6%
4.	Inception-ResNet V4	94.3%	94.1%	93.2%	94.2%
5.	VGG 16	91.5%	91.6%	90.5%	91.4%
6.	Xception	87.2%	87.3%	86.7%	86.3%

**Table 4:** Performance of the ensemble learning model for classification.

Sl. No	Ensemble ML Models	Accuracy	Precision	Recall	F1 Score
1.	Random Forest	82.4%	82.7%	83.6%	81.5%
2.	Gradient boosting	80.9%	80.3%	80.88%	79.5%
3.	AdaBoosting	89.4%	88.5%	89.1%	87.6%
4.	XGBoosting	79.5%	80.2%	79.8%	80.3%

**Table 5:** Improved performance with hyperparameter tuning techniques.

Sl. No	Ensemble ML Models	Accuracy	Precision	Recall	F1 Score
1.	Random Forest	86.3%	86.3%	87.9%	86.2%
2.	Gradient boosting	82.7%	82.7%	83.7%	81.3%
3.	AdaBoosting	91.37%	91.87%	90.3%	91.78%
4.	XGBoosting	80.34%	81.67%	80.48%	81.78%

## Data Availability

Dataset is available on request.
